# The influence of haemoglobin and iron on *in vitro* mycobacterial growth inhibition assays

**DOI:** 10.1038/srep43478

**Published:** 2017-03-03

**Authors:** Rachel Tanner, Matthew K. O’Shea, Andrew D. White, Julius Müller, Rachel Harrington-Kandt, Magali Matsumiya, Mike J. Dennis, Eneida A. Parizotto, Stephanie Harris, Elena Stylianou, Vivek Naranbhai, Paulo Bettencourt, Hal Drakesmith, Sally Sharpe, Helen A. Fletcher, Helen McShane

**Affiliations:** 1 The Jenner Institute, University of Oxford, Oxford, UK; 2 Public Health England, Porton Down, Salisbury, UK; 3 Weatherall Institute of Molecular Medicine, University of Oxford, Oxford, UK; 4 London School of Hygiene and Tropical Medicine, London, UK

## Abstract

The current vaccine against tuberculosis, live attenuated *Mycobacterium bovis* BCG, has variable efficacy, but development of an effective alternative is severely hampered by the lack of an immune correlate of protection. There has been a recent resurgence of interest in functional *in vitro* mycobacterial growth inhibition assays (MGIAs), which provide a measure of a range of different immune mechanisms and their interactions. We identified a positive correlation between mean corpuscular haemoglobin and *in vitro* growth of BCG in whole blood from healthy UK human volunteers. Mycobacterial growth in peripheral blood mononuclear cells (PBMC) from both humans and macaques was increased following the experimental addition of haemoglobin (Hb) or ferric iron, and reduced following addition of the iron chelator deferoxamine (DFO). Expression of Hb genes correlated positively with mycobacterial growth in whole blood from UK/Asian adults and, to a lesser extent, in PBMC from South African infants. Taken together our data indicate an association between Hb/iron levels and BCG growth *in vitro*, which may in part explain differences in findings between whole blood and PBMC MGIAs and should be considered when using such assays.

Tuberculosis (TB) remains a serious global health and socioeconomic threat, with 9.6 million new cases and 1.5 million deaths per year^1^. The only currently available vaccine, BCG, has poor efficacy against adult pulmonary disease in the tropics, where TB incidence is greatest^2^. There is an urgent need for a new vaccine, but successful development is hampered by the lack of an immune correlate of protection^3^. Unlike relying on individual immune parameters, mycobacterial growth inhibition assays (MGIAs) are functional assays that take into account a whole range of immune mechanisms and their interactions. Such assays have been described by Hoft, Morris and others^4^,^5^ and recently reviewed^6^. The ‘MGIT’ assay, originally developed by Wallis *et al*., involves the co-culture of whole blood, or isolated cells, with mycobacteria for a 72–96 hour period, followed by quantification of remaining mycobacteria using the BD Bactec MGIT system^7^. A colony-forming unit (CFU)-based net growth value calculated relative to a control and stock standard curve permits measurement of mycobacterial growth. This assay has been adapted for measuring vaccine effect in whole blood and peripheral blood mononuclear cells (PBMC) from humans^8^ and in splenocytes from mice^9^,^10^. Here we show that there is no correlation between mycobacterial growth in human whole blood and PBMC MGIT assays, and investigate haemoglobin (Hb) and iron as potential contributing factors to this discrepancy.

Iron is required for the growth and survival of most intracellular bacteria. There is a considerable body of literature demonstrating the importance of iron for the growth and pathogenesis of *Mycobacterium tuberculosis (M.tb*). Early work by Kochan *et al*. showed that *M.tb* bacilli can only grow in serum with a sufficient concentration of iron^11^. Addition of iron increases both intra- and extra-cellular mycobacterial growth *in vitro*^12^,^13^; effects that are prevented by exposure to iron chelating agents^13^. *In vivo*, increased iron results in decreased resistance to TB disease and worse clinical outcome^14^. Furthermore, it has been suggested that iron overload contributes to TB susceptibility in Africa^15^,^16^,^17^, and correction of iron overload in mice eliminates this effect^18^. Siderophore-mediated iron uptake pathways in mycobacteria are well-characterised, and allow the pathogen to remove iron from human transferrin and lactoferrin and transport it to mycobactins in the cell wall or the iron transport system^19^. However, synthesis of the molecules involved is metabolically costly to the pathogen, and transferrin accounts for less than 1% of the body’s total iron^20^.

The majority of dietary iron in the host (~70–80%) is stored in the form of heme: primarily as haemoglobin^21^. Many bacteria, both Gram-negative and Gram-positive, are known to use heme as a major source of iron^22^,^23^,^24^. There is now a growing body of evidence indicating a heme iron uptake pathway in mycobacteria. Jones and Niederweis demonstrated that the growth defect of a siderophore-deficient strain of *M.tb* is rescued by the addition of heme^25^. Addition of exogenous heme to an *M.tb* mutant with an interrupted heme biosynthetic pathway restores growth^26^, and addition of haemoglobin increases mycobacterial growth *in vitro*^12^. Furthermore, a gallium-substituted heme derivative is toxic to mycobacterial cells, suggesting that it is taken up by the mycobacteria, and may be used in the cell wall environment or broken down in the cytoplasm^27^. In 2011, it was demonstrated that *M.tb* can utilise heme from haemoglobin, and the pathway by which it does so has since been characterised^28^. To date, four members of the host-derived heme uptake pathway have been described, including Rv0203 and MhuD, the mycobacterium heme degrader which catalyses the final step of heme acquisition and degradation to iron and by-products^29^,^30^,^31^,^32^.

We have evaluated the effect of Hb and iron on mycobacterial growth *in vitro* using the previously-described MGIT assay^7^,^8^,^9^,^10^. This was investigated across different species commonly used in TB vaccine testing; human, mouse and non-human primates (NHPs). We explored the relationship between Hb and mycobacterial growth in a previously reported trial of BCG vaccination in healthy UK volunteers^8^, and report a correlation between mean corpuscular Hb and *in vitro* growth of BCG in human whole blood. Experimental addition of Hb or ferric iron resulted in increased mycobacterial growth, whereas addition of the iron chelator deferoxamine reduced it. Expression of Hb complex genes correlated with mycobacterial growth in whole blood from UK/Asian adults and PBMC from South African infants. Our data indicate an association between Hb/iron levels and BCG growth *in vitro*, which may be a confounding factor and contribute to variability when using whole blood, and to a lesser extent, PBMC mycobacterial growth inhibition assays in vaccine and mycobacterial immunological studies.

## Results

### No association between mycobacterial growth in the whole blood and PBMC MGIT assays

**Figure 1:**
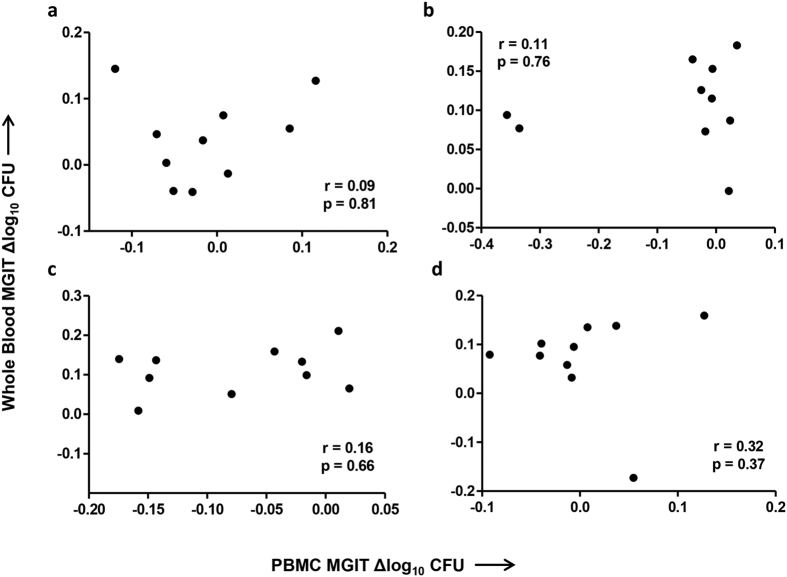
No correlation between *in vitro* mycobacterial growth in whole blood and PBMC. Spearman’s correlation between BCG Pasteur growth in the whole blood and PBMC MGIT assays in 10 healthy human volunteers at (**a**) week 0, (**b**) week 4, (**c**) week 8 and (**d**) week 24 following BCG vaccination. Previously BCG vaccinated and naïve volunteers were grouped together. Points represent the mean of duplicate cultures. Δ log_10_ CFU = (log_10_ CFU of sample − log_10_ CFU of control).

Where data was available from MGIT assays performed using PBMC and separately whole blood from the same individuals from the previously reported BCG vaccination study in UK adults^8^ (n = 10), correlations were performed between outcomes of the two assays. As the same pattern was observed for BCG naïve and historically vaccinated individuals, these groups were combined. The correlation was not significant at any time-point (p = 0.81, p = 0.76, p = 0.66 and p = 0.37, Spearman’s correlation, Fig. 1a–d), or when all time-points were combined (r = 0.06, p = 0.71, Spearman’s correlation). A second experiment was performed in a validation cohort of 12 healthy UK volunteers at baseline. Again there was no correlation between mycobacterial growth in whole blood and PBMC from the same individuals (r = −0.14, p = 0.67, Spearman’s correlation, data not shown). There was no statistically significant difference between a linear mixed model of whole blood MGIT~PBMC MGIT including or excluding the effect of MCH or Hb (when time-point was included as an interaction term).

### Mean corpuscular Hb correlates with mycobacterial growth in the human whole blood MGIT assay

**Figure 2:**
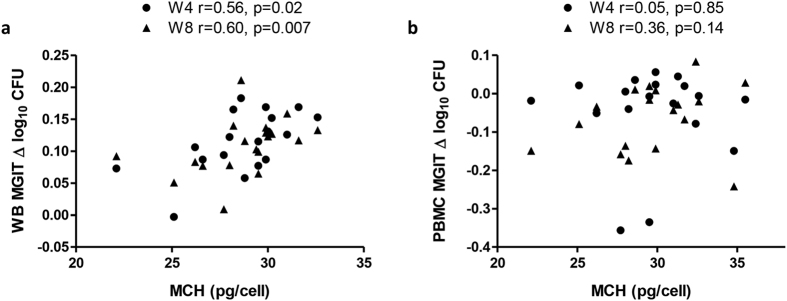
Correlation between MCH and mycobacterial growth in the whole blood MGIT assay. Spearman’s correlations between mean corpuscular haemoglobin (MCH) at baseline and *in vitro* BCG Pasteur growth in (**a**) whole blood and (**b**) PBMC of 19 healthy UK adult volunteers at 4 weeks (circles) and 8 weeks (triangles) following BCG vaccination. Previously BCG vaccinated and naïve volunteers were grouped together. Points represent the mean of duplicate cultures. Δ log_10_ CFU = (log_10_ CFU of sample − log_10_ CFU of control).

Mean corpuscular haemoglobin (MCH) and Hb concentration at baseline from volunteers in the human BCG vaccine study were related to subsequent MGIT mycobacterial growth in whole blood and PBMC where both measures were available (n = 19 and n = 18 respectively). MCH correlated significantly with MGIT mycobacterial growth in whole blood taken at 4 and 8 weeks post-BCG vaccination (p < 0.05 and p < 0.01 respectively, Spearman’s correlation, Fig. 2a). When MCH was added as a continuous variable in a linear model of whole blood MGIT ~ MCH, MGIT variance was significantly explained by MCH at week 4 (r^2^ = 0.33, p < 0.05) and trending towards significance at week 8 (r^2^ = 0.19, p = 0.06). This association was lost when the MGIT assay was performed using PBMC (p = 0.4 and p = 0.3 respectively, Spearman’s correlation, Fig. 2b). The correlation between Hb concentration and mycobacterial growth was not statistically significant (data not shown).

### Mycobacterial growth and Hb concentration decrease following BCG vaccination and successive bleeds in Rhesus macaques

**Figure 3:**
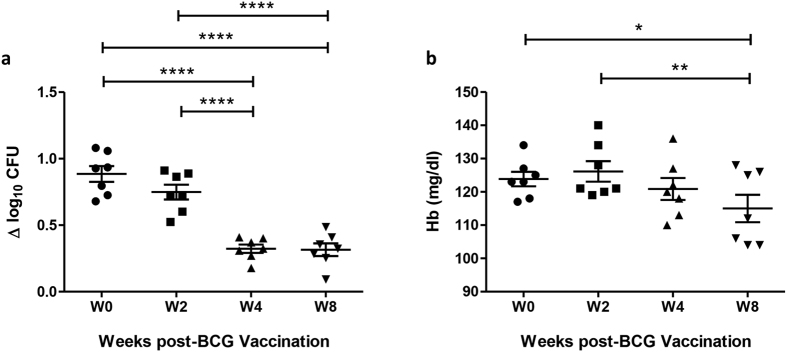
Reduction in Mycobacterial growth and Hb concentration following BCG vaccination and successive bleeds in Rhesus macaques. (**a**) The MGIT assay was performed using BCG Pasteur and (**b**) Hb measured pre- and post-BCG vaccination using whole blood from 7 Rhesus macaques. Points represent the mean of duplicates from individual animals and bars represent the mean values with SEM. Shapes represent different time-points. Having passed a normality test, a repeated measures ANOVA was performed followed by a Bonferroni post-test where *represents a p-value of <0.05, **represents a p value of <0.005, and ****represents a p-value of <0.0001. Δ log_10_ CFU = (log_10_ CFU of sample − log_10_ CFU of control).

In a study of 7 healthy Rhesus macaques who received primary BCG vaccination, bleeds of ~7.5% total blood volume (TBV) were taken at screening, baseline and 2, 4 and 8 weeks post-vaccination. Mycobacterial growth as measured by the whole blood MGIT assay decreased significantly at weeks 4 and 8 following BCG vaccination compared with baseline (p < 0.0001, Repeated measures ANOVA, Fig. 3a), coincident with a decrease in Hb concentration at week 4 and significantly at week 8 relative to baseline (p < 0.05, Repeated measures ANOVA, Fig. 3b). Measures of Hb concentration were used, as MCH or other haematological parameters were not available for these studies. Using the definition of anaemia in macaques of Adams *et al*. (Hb concentration >2 standard deviations below the baseline mean)^33^, 4 out of 7 (57%) of animals were mildly anaemic at 8 weeks. Consistent with an absence of association between Hb concentration and mycobacterial growth in humans, the correlation between Hb and mycobacterial growth in macaques was not statistically significant (data not shown). When Hb was added as a continuous variable in a linear mixed model of MGIT ~ Hb + Day while accounting for random effects of the animals, it did not contribute significantly to MGIT assay variance (p = 0.6).

### Addition of haemoglobin to PBMC MGIT cultures results in increased mycobacterial growth

**Figure 4:**
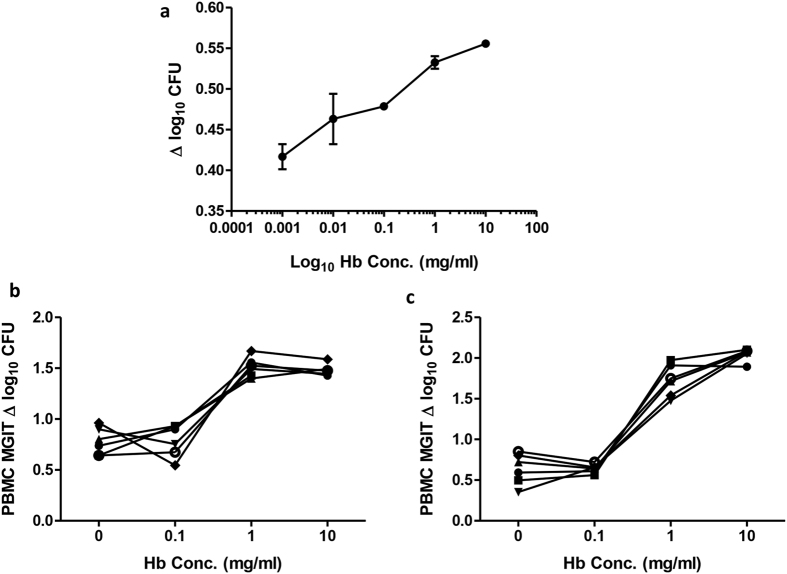
Hb concentration influences *in vitro* mycobacterial growth. BCG Pasteur growth was measured at increasing concentrations of Hb added (**a**) directly to Bactec MGIT tubes, and to 96 hour cultures of PBMC from (**b**) 6 human volunteers or (**c**) 6 macaques. Lines represent individuals; points represent the mean of duplicates and bars the SEM. Δ log_10_ CFU = (log_10_ CFU of sample − log_10_ CFU of control).

To further investigate the effect of Hb concentration on mycobacterial growth *in vitro*, Hb was titrated either directly into Bactec MGIT tubes (Fig. 4a) or into 96 hour MGIT cultures of PBMC from 6 human volunteers (Fig. 4b) or 6 Rhesus macaques (Fig. 4c). In all cases, a higher concentration of Hb was associated with increased mycobacterial growth. These results were confirmed in two further experiments using human PBMC, and a similar pattern was observed using mouse splenocytes (n = 6, data not shown). The effect was greater when cells were present than not (Δ log_10_ CFU between 0 mg/ml and 10 mg/ml = 0.7 and 0.3 respectively). It was not possible to compare the slopes of the lines as each had a different fit, with a linear response in the direct-to-MGIT experiment and a three-parameter dose-response curve plateauing at 1 mg/ml in cells.

### Addition of the iron chelator deferoxamine (DFO) results in decreased mycobacterial growth

**Figure 5:**
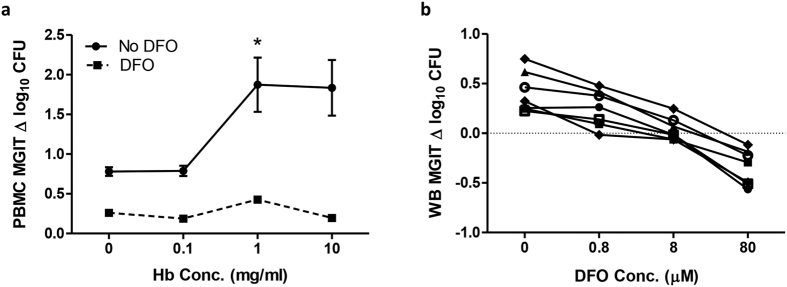
Addition of iron chelator negates the effect of Hb concentration on *in vitro* mycobacterial growth. (**a**) BCG Pasteur growth was measured following 96 hour co-culture with human PBMC with the addition of increasing concentrations of Hb, with (dotted line) or without (solid line) treatment with 8 μM DFO. Points represent the mean of 6 individuals and bars the SEM. There was a clear reduction in mycobacterial growth at all concentrations following treatment with DFO. There was a significant increase in mycobacterial growth between 0.1 mg/ml and 1 mg/ml Hb in the absence of DFO (p < 0.05, paired t-test), but addition of DFO removed this effect. (**b**) BCG Pasteur growth was measured following 96 hour co-culture with whole blood from 6 human volunteers with the addition of increasing concentrations of DFO. Lines represent individuals; points represent the mean of duplicates. Δ log_10_ CFU = (log_10_ CFU of sample - log_10_ CFU of control).

To explore the contribution of iron to the effects of Hb on mycobacterial growth, the iron chelator deferoxamine (DFO) was added at a concentration of 8 μM alongside increasing concentrations of Hb into 96 hour MGIT cultures of PBMC from 6 human volunteers. Addition of DFO resulted in a clear reduction in mycobacterial growth at all concentrations following treatment with DFO. In accordance with the preceding experiments, there was a significant increase in mycobacterial growth between 0.1 mg/ml and 1 mg/ml Hb in the absence of DFO (p < 0.05, paired t-test, Fig. 5a), but addition of DFO negated this effect (Fig. 5a). When DFO was titrated in the MGIT assay using whole blood from 7 healthy human volunteers (where haemoglobin was expected to be within the normal physiological range), an increased concentration of DFO was associated with decreased mycobacterial growth (Fig. 5b).

### Addition of iron results in increased mycobacterial growth

**Figure 6:**
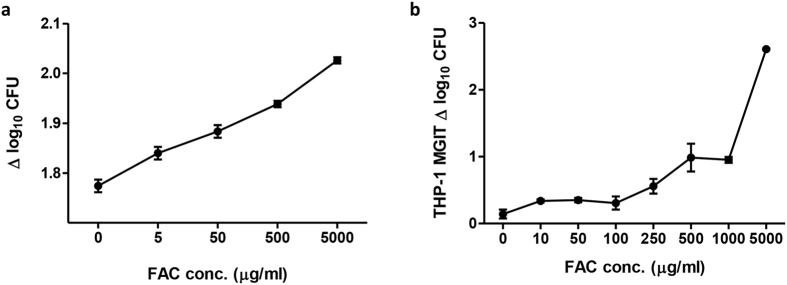
Addition of iron enhances *in vitro* mycobacterial growth. BCG Pasteur growth was measured at increasing concentrations of ferric ammonium citrate (FAC) added (**a**) directly to Bactec MGIT tubes and (**b**) to 96 hour cultures of THP-1 cells. Points represent the mean of duplicates and bars the SEM. Δ log_10_ CFU = (log_10_ CFU of sample − log_10_ CFU of control).

To further explore the influence of iron, ferric ammonium citrate (FAC) was titrated either directly into Bactec MGIT culture tubes containing BCG Pasteur (Fig. 6a) or to 96 hour MGIT cultures of human THP-1 cells (Fig. 6b). In both cases, an increased concentration of FAC was associated with increased mycobacterial growth. Once again, this effect was greater when cells were present than not (Δ log^10^ CFU between 0 μg/ml and 5000 μg/ml = 2.5 and 0.3 respectively), with a significant difference between the slopes of the lines (p < 0.0001). In a study of 7 Cynomolgus macaques bled on 3 successive occasions at fortnightly intervals with no intervention, there were no significant changes in mycobacterial growth or Hb concentration. However, addition of Hb or FAC to the whole blood MGIT cultures increased mycobacterial growth whereas adding DFO significantly reduced mycobacterial growth (p < 0.005, paired t-test on AUC, Table 1).

### Expression of Hb genes correlates with mycobacterial growth in both the whole blood and PBMC MGIT assays

**Figure 7:**
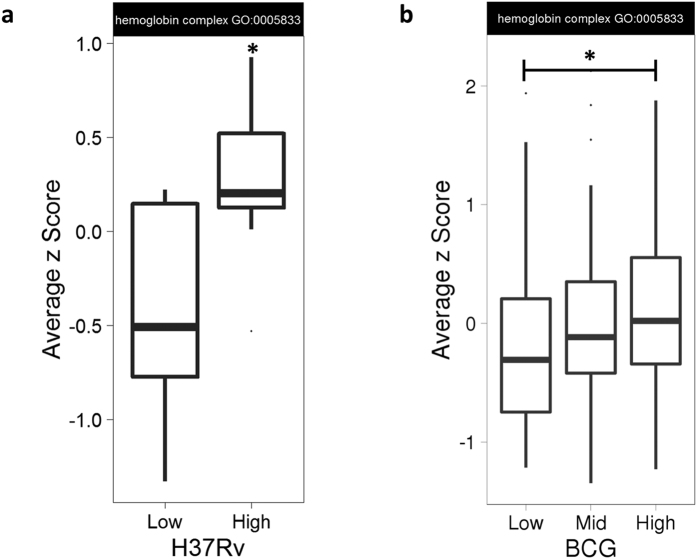
Increased expression of Hb complex genes is associated with increased *in vitro* mycobacterial growth. Individuals were stratified by mycobacterial growth (low and high or low, mid and high Δ log_10_ CFU) from two different studies of (**a**) whole blood from 21 healthy adults from the UK/Asia in an *M.tb* H37Rv MGIT assay and (**b**) PBMC from 130 South African infants in a BCG Pasteur MGIT assay. Gene expression levels were taken from unstimulated samples and the z-score averaged across the 11 gene members of the GO category ‘haemoglobin complex’. The box represents the upper and lower quartiles and the line the median value. The whiskers represent the maximum and minimum values excluding outliers. A Mann-Whitney U test was performed between groups where *represents a p-value of <0.05.

Expression of the 11 genes classified in the Gene Ontology (GO) category ‘haemoglobin complex’ (GO:0005833) was measured by microarray in unstimulated whole blood from 21 healthy UK/Asian adults and PBMC from 130 South African infants. MGIT assays were performed using either *M.tb* H37Rv (in whole blood assays) or BCG Pasteur (in PBMC assays) for *in vitro* infections. Gene set enrichment analysis was performed on this GO category, where genes were ranked according to MGIT log_2_ fold change (the amount by which gene expression increases per unit increase in mycobacterial growth), which gave a p-value of 0.015 and 2.19e-14 for the UK/Asian and the South African cohort respectively. To visualise the association, Z-scores were averaged across the genes in this category to give a single score per individual and individuals were stratified by mycobacterial growth into two or three quantiles (low and/or mid and high Δ log_10_ CFU). There was a significant difference in Hb gene expression between groups with ‘high’ and ‘low’ mycobacterial growth in whole blood (p = 0.015, Mann-Whitney U test, Fig. 7a), and to a lesser extent PBMC (p = 0.035, Mann-Whitney U test, Fig. 7b). Differential expression estimates of individual Hb genes in units of mycobacterial growth are shown in Table 2. Genes that were expressed below the limit of detection were excluded from the analysis.

## Discussion

We have previously shown that the *in vitro* mycobacterial growth indicator tube (MGIT) assay is able to detect enhanced mycobacterial growth inhibition following primary BCG vaccination in human UK volunteers^8^. A stronger effect was observed when using cryopreserved PBMC compared with whole blood, with a significant difference at both 4 and 8 weeks post-BCG detected using PBMC but only at 8 weeks using whole blood (the latter was not significant following correction for multiple comparisons). A difference between naïve and historically BCG-vaccinated individuals at baseline was observed using the PBMC but not whole blood assay^8^. When mycobacterial growth in whole blood and PBMC cultures was compared in this and a further validation cohort, there was no association between outcomes of the two assays. One of the potential explanations is the presence of haemoglobin (Hb) in whole blood but not PBMC. That there remained no correlation between the whole blood and PBMC MGIT assays following statistical correction for either Hb or mean corpuscular Hb (MCH) in whole blood indicates the contribution of other additional factors. There are several components present in whole blood but not PBMC which have previously been shown to impact mycobacterial growth *in vitro* and may contribute to these discrepancies including neutrophils^34^, antibodies^35^,^36^ and serum constituents such as complement^37^.

A correlation was observed between mean corpuscular haemoglobin (MCH) at baseline and BCG growth using the whole blood MGIT assay in the same cohort. It is probable that the lack of association with the MGIT assay at baseline or week 24 is due to small sample size and noise in the assay, as measures of Hb were unlikely to alter significantly over the course of this study given its non-intensive bleed schedule. Clinical data was available only at baseline when volunteers were screened for eligibility and therefore not matched by time-point. The relationship with MCH is lost when using the PBMC-based assay, which may be expected due to the absence of erythrocytes and therefore Hb in PBMC. Interestingly, the correlation between mycobacterial growth and Hb itself was not significant. It is possible that MCH represents a more sensitive measure, as it also takes into account the number of red blood cells and is more sensitive in the detection of iron-deficiency anaemia^38^,^39^. Alternatively, other stores of iron may be considerably impaired while Hb remains high, with iron disproportionately channelled to erythrocytes to compensate for a deficit in other parts of the body. However, that Hb and MCH have an almost significant correlation in this study (Spearman’s, p = 0.07, data not shown) suggests that mycobacterial growth may also be associated with Hb given a larger sample size.

Given this relationship, the reduction in mycobacterial growth in whole blood that was observed following BCG vaccination in macaques may have been influenced by a concurrent reduction in Hb concentration due to repeated bleeds. However, the effect of BCG vaccination remained significant at weeks 4 and 8 following statistical correction for changes in Hb concentration, suggesting that a vaccine effect is still driving the majority of the response. This is consistent with a previous study in Cynomolgus macaques in which mycobacterial growth inhibition in the whole blood MGIT assay was enhanced following BCG vaccination when no change in Hb was induced (Tanner *et al*. unpublished data), and to our knowledge is the first report in the literature of an MGIA using NHP samples. Ideally, a matched control group of macaques undergoing the same bleed schedule but not receiving BCG vaccination would have allowed determination of the effect of repeated bleeds and Hb alone. However, to remain within the guidelines for a study involving no intervention, it was not possible to collect blood from animals in a follow-up study of a sufficient volume and frequency to induce a significant drop in Hb.

To further investigate the effect of Hb on the growth of BCG in this assay, Hb was experimentally manipulated by addition at increasing concentrations directly to BCG-inoculated MGIT tubes or to 96 hour cultures of PBMC from humans and NHPs. In all cases, higher concentrations of Hb resulted in increased mycobacterial growth. However, the effect in cells plateaued at 1 mg/ml; which may be due to biological saturation as the mycobacteria sate their requirements and stores, or a technical effect of the dark pigment on the ability of the MGIT system to detect fluorescence. It was hypothesised that the effect of Hb on mycobacterial growth was mediated by iron, as confirmed by addition of the iron chelator DFO. The fact that DFO did not entirely negate the effect of increasing Hb concentration suggests that other constituents of Hb, such as amino acids, may mediate a minor effect, or that the concentration of DFO was not sufficiently high to chelate all of the iron. Cell viability studies indicated that a higher concentration of DFO would be detrimental to cell survival so this could not be applied. A limitation of these experiments is that even at low concentrations, DFO could have some toxicity and affect cellular functions independent of iron, and may impact anti-microbial functions. Another factor that may contribute to the effect of Hb in this model is nitric oxide (NO). There is a substantial body of evidence indicating a role for NO in host-mediated antimycobacterial activity in mice, and to a lesser extent, humans^40^. Human Hb, like bacterial flavohaemoglobin, exhibits distinct nitric oxide dioxygenase (NOD) activity, which may act to reduce the toxic effects of exogenous NO resulting from the innate immune response^41^,^42^.

The contribution of iron was further supported by increased mycobacterial growth resulting from addition of iron, both directly to MGIT tubes and in 96 hour cultures of THP-1 cells. Interestingly the effect of adding iron, and to a lesser extent Hb, was stronger when cells were present than not. This suggests that iron is influencing the cells as well as the mycobacteria resulting in an additive effect, the mechanism for which warrants further study. In a study of 7 Rhesus macaques, 3 sequential bleeds of 20 ml were taken (the maximum permissible in a study with no intervention due to ethical regulations). As a reduction in Hb was not observed, the effect of this in the absence of vaccination could not be assessed. However, in support of the previous findings, experimental addition of Hb and iron both increased mycobacterial growth *in vitro* at all time-points in this study, and addition of the iron chelator DFO significantly reduced growth compared with the control group undergoing a standard MGIT culture. This body of data is consistent with literature on iron and mycobacteria, which demonstrates the importance of iron for growth and pathogenesis^11^,^12^,^13^,^14^,^15^,^16^,^17^,^18^.

When Hb genes were considered, there was a significant correlation between gene expression and mycobacterial growth in whole blood, and to a lesser extent PBMC. Hb complex genes were averaged to create an overall score, demonstrating significantly higher expression in the group of individuals with high mycobacterial growth compared to those with low mycobacterial growth. This was consistent across two different cohorts using two different mycobacterial strains in two different compartments, further supporting a role for Hb in influencing mycobacterial growth. Although an association in PBMC is perhaps surprising, the most likely source of globin mRNA is circulating CD34+ immature erythroblasts, which though present in small numbers in this compartment will express high levels of globin genes^43^. Higher frequencies of such cells are present in whole blood, which may contribute to the stronger correlation.

Though the role of iron in mycobacterial growth is well-known, the effect of varying levels *in vivo* on functional *in vitro* growth assays has not been previously addressed. This represents an important potential confounding factor and source of variability in the whole blood MGIT assay in particular that may contribute in part to its reduced ability to detect a vaccine-induced response. The reduction in Hb observed following sequential bleeds in NHPs may influence functional assays in time-course experiments and would likely apply to other small animal studies of sequential sampling. Although humans are more resistant to such perturbations due to their relative size, there is evidence for Hb deferral, iron depletion and iron deficiency in individuals who frequently donate blood^44^,^45^,^46^. In a study of reference ranges for people aged 6 months to 74 years in the US, Hb concentration varied by as much as 64 mg/ml between individuals^47^, whereas we have shown that a difference of just 1 mg/ml can have a significant effect on mycobacterial growth (Fig. 4).

Multiple factors contribute to such variability. It is now widely recognised that Hb concentration has some degree of genetic basis^48^,^49^ and differs considerably by race^47^,^50^,^51^. Infections and underlying health issues such as sickle cell anaemia and malaria can affect both Hb concentration and availability of ‘free’ haemoglobin due to haemolysis^52^,^53^. Hb levels are also influenced by age, sex and external factors including nutritional status, certain medications, altitude, and tobacco smoking^47^,^52^,^54^,^55^,^56^. Furthermore, there is evidence for an effect of sleep and circadian rhythms, with Hb concentration fluctuating over a 24 hour period^57^. Interestingly a recent study reported that Hb concentration in infants was associated with time of vaccination with BCG and/or DTP^58^. In the South African infant correlates of risk study described here, none of the Hb complex genes were differentially expressed between unstimulated and BCG stimulated samples, which does not support an effect of BCG vaccination on Hb (data not shown).

In addition to Hb, it would be prudent to take into account any variation in iron status when comparing different populations or demographics in functional assays. In most countries of sub-Saharan Africa, anaemia (due to malnutrition, malaria and helminth infections) is considered a widespread and serious public health problem^59^. The leading cause of iron deficiency worldwide is acute or chronic blood loss, with women of fertile age at highest risk due to menstrual losses^60^. Iron levels vary by sex and cyclically in females; approximately 5% of menstruating women report suffering from menorrhagia which frequently results in totally depleted iron stores^61^. Variations in Hb or iron levels are unlikely to impact drug studies using the MGIT assay, where bactericidal activity is pronounced, or investigations of TB patients where clinical parameters form part of the overall picture. However, in vaccine studies with a small magnitude of effect where physiological variations are confounding, such differences may be problematic.

The observation that Hb is associated with increased mycobacterial growth *in vitro* may have wider implications for the pathogenesis of *M.tb* and the efficacy of BCG vaccination *in vivo*. It is possible that high Hb availability could predispose to exacerbated TB disease or increased susceptibility, as shown in the case of iron overload^15^. Although low Hb levels are predictive of incident TB, this is likely reverse causality, as anaemia is a common haematological abnormality in TB patients, and typically resolves following anti-TB treatment^62^,^63^. The lower Hb and iron levels prevalent in sub-Saharan Africa (due to a combination of factors including genetics and malnutrition)^59^ may contribute to the poor protective efficacy of BCG vaccination in this population if BCG is unable to replicate efficiently, while haemolysis-inducing diseases could act to boost BCG growth and immunogenicity by increasing availability of free Hb. To our knowledge there are no studies addressing TB susceptibility in malaria-infected individuals, although interestingly placental malaria has been associated with an attenuated immune response to BCG vaccination at birth^64^. There is one report of significantly increased rates of lymph node TB but reduced rates of pulmonary TB in patients with sickle cell disease^65^. Boelaert *et al*. suggest that iron status, which also affects immune regulation, should be taken into account in further studies of anti-*M.tb* vaccines, for example comparing results of vaccinating animals fed on iron-poor vs. iron-enriched diets^66^. We propose that similar animal experiments and human epidemiological analysis would be of use investigating the influence of Hb levels on BCG vaccine efficacy.

In conclusion, we have demonstrated that BCG, like *M.tb*, is able to utilise iron for growth *in vitro* and that increased haemoglobin results in increased BCG growth; in part due to bound iron. It is likely that BCG has a mechanism similar to *M.tb* allowing it to sequester heme-bound iron, although to our knowledge this has not been previously described. The fact that the MGIT assay is able to discriminate between individuals with different Hb levels (even within the normal range) is an indicator of the sensitivity of the assay and its ability to detect differences in mycobacterial growth under slightly altered culture conditions. We have identified two mechanisms by which variability in Hb and iron may impact mycobacterial growth in this assay: inter-individual variability within human and animal populations and intra-individual variability within animal models over a time-course. We suggest that vaccine response measurements made using whole blood MGIAs in particular should be interpreted with caution. This principle may have wider applicability across other blood-based functional bactericidal assays, as iron is a requirement for the growth and survival of many pathogens including *P.falciparum, E.coli* and *S.aureus*^22^,^23^,^24^. Saturating iron without reaching toxic levels or chelating iron followed by adding in a predefined amount are potential methods for controlling for this variable, though the impact is likely to be biologically far-reaching and complex. A PBMC- or splenocyte-based assay could represent an alternative that reduces such confounders, though it should be noted that a weak association was still observed between mycobacterial growth and Hb gene expression in the infant PBMC study described. The fact that monocytes are a major iron store^67^ may in part explain the importance of the frequency of this cell type in determining mycobacterial growth in the PBMC MGIT assay^68^.

## Methods

### Human studies

All methods used in the human studies described were carried out in accordance with the ethical principles set forth in the Declaration of Helsinki as agreed by the World Medical Association General Assembly (Washington 2002), ICH Good Clinical Practice (GCP) and local regulatory requirements.

### Human BCG study

The details of the study design and participants have been described elsewhere^8^. Briefly, this was a non-randomised clinical study in healthy adults with or without a history of BCG vaccination. Volunteers were aged between 18 and 46 years with no evidence of latent *M.tb* infection. All individuals received a single intradermal dose of 2–8 × 10^5^ CFU of BCG SSI and follow-up bleeds were taken at 4, 8 and 24 weeks post-vaccination. The study was approved by the Oxfordshire Research Ethics Committee (OxREC A) and written informed consent was obtained from all individuals prior to enrolment in the trial.

### Adult whole blood gene expression study

21 healthy adults were recruited with or without a history of BCG vaccination. Volunteers were aged between 18 and 56 years with no past history of TB or evidence of latent infection. 6 were European and 15 were Asian of Indian sub-continent ethnicity. Peripheral blood from each volunteer was collected in a PAXgene^©^ tube and stored in accordance with the manufacturer’s instructions for later microarray transcriptomic studies. The study was approved by the Ministry of Defence Research Ethics Committee, and written informed consent was obtained from all individuals prior to enrolment in the trial.

### Infant correlates of risk study

The infant correlates of risk cohort used in this study formed part of a larger MVA85A Phase IIb efficacy trial (ClinicalTrials.gov number NCT00953927); the details of which have been described elsewhere^69^. Briefly, this was a double-blind, randomised, placebo-controlled trial. 2797 healthy South African infants were enrolled (aged 4–6 months) who had received BCG vaccination within seven days of birth. Infants were randomised to receive either MVA85A vaccination (n = 1399) or an equal volume of Candida skin test antigen (n = 1398), and actively followed up every 3 months for up to 37 months. The primary study outcome was safety; the primary efficacy endpoint was incident tuberculosis. MVA85A did not confer additional protection against TB disease in this trial beyond that conferred by BCG alone (VE 17.3%; 95% CI −31.9 to 48.2)^69^. The correlates of risk study was conducted on PBMC and serum taken at enrolment (4–6 months) and D28 post-boost (n = 258) and has been previously described^70^. Multiple assays were run with a priority listing^71^; the MGIT assay was performed only when a sufficient number of cells were available, and samples were excluded if the standard deviation between duplicates was greater than 40 hours. This resulted in data available from 116 infants. To avoid potential confounding effects of MVA85A vaccination, data used for this analysis was from the enrolment sample only (BCG vaccinated but pre-MVA85A). The trial was approved by the University of Cape Town Faculty of Health Sciences Human Research Ethics Committee, Oxford University Tropical Research Ethics Committee and the Medicines Control Council of South Africa, and informed consent was obtained from the mothers of all the infants prior to enrolment in the trial.

### Non-human primate studies

All animal procedures and study design were approved by the Public Health England, Porton Down Ethical Review Committee, and authorised under an appropriate UK Home Office project licence. All methods were performed in accordance with the relevant guidelines and regulations including the UK Animals (Scientific) Procedures Act 1986 (ASPA), the Code of Practice for the housing and Care of Animals Bred, Supplied or Used for Scientific Purposes (December 2014); the NC3Rs; and the Guidelines on Primate Accommodation, Care and Use (NC3Rs, 2006). The animals used in the BCG vaccination study were 7 Rhesus macaques of Indian genotype aged between 9 and 12 years. The animals used in the Hb study were 7 Cynomolgus macaques of Mauritian genotype aged between 12 and 16 years. Animals were obtained from established UK breeding colonies and remained in the colony in their established socially compatible groups. For procedures requiring removal from their housing, animals were sedated by intramuscular injection with ketamine hydrochloride (10 mg/kg body weight) (Ketaset, Fort Dodge Animal Health Ltd. UK). None of the animals had previous exposure to mycobacterial antigens (as confirmed by a negative tuberculin skin test and *ex-vivo* IFN-γ ELISpot for PPD, ESAT-6 and CFP-10). Animals were monitored daily for behavioural or clinical changes, and throughout the duration of the studies for weight, temperature, lymph node size and Hb levels. Rhesus macaques were vaccinated using an adult human dose of BCG Danish strain 1331 (SSI, Copenhagen) 100 μl of 2–8 × 10^6^ CFU/ml intradermally in the upper left arm using a limited volume insulin syringe.

### Bacterial strains and culture

BCG Pasteur was obtained from PHE (Porton Down, UK) and Aeras (MD, USA). *M.tb* H37Rv was obtained from BEI Resources (VA, USA). PHE BCG and *M.tb* H37Rv stock vials were cultured in 6 Bactec MGIT tubes for 8 days and cultures were then pooled, aliquoted and frozen at −80° as previously described^7^. Aeras BCG was used directly from a thawed stock vial.

### MGIT Mycobacterial growth inhibition assay

The whole blood mycobacterial growth inhibition assay was performed based on the previously described methods of Wallis *et al*.^7^. Duplicate tubes containing 300 μl of whole blood were incubated on a 360° rotator at 37 °C with 300 μl of RPMI-MGIT (RPMI containing 10% PHS, 2 mM l-glutamine and 25 mM HEPES) seeded with ~150 CFU BCG Pasteur or *M.tb* H37Rv for 96 hours. Cells were then lysed with sterile water, and the lysate transferred to a Bactec MGIT tube supplemented with PANTA antibiotics and OADC enrichment broth (Becton Dickinson, UK). Tubes were placed on the BACTEC 960 machine (Becton Dickinson, UK) and incubated at 37 °C until the detection of positivity by fluorescence. On day 0, duplicate direct-to-MGIT viability control tubes were set up by inoculating supplemented BACTEC MGIT tubes with the same volume of mycobacteria as the samples. MGIT assays using cryopreserved PBMC and mouse splenocytes were performed as previously described^8^,^9^. Methods are as for the whole blood assay, replacing 300 μl of whole blood with 1 × 10^6^ cryopreserved PBMC (thawed as previously described^71^ and rested for 2 hours) in 300 μl of RPMI-MGIT per culture. For the iron supplementation experiment, the human acute monocytic leukaemia cell line THP-1 (ATCC TIB202) was used. Differentiation into macrophage-like cells was induced with 20 nM phorbol 12-myristate 13-acetate (PMA) overnight. Differentiated cells were maintained adherent in 24-well plates in R10 medium prior to infection with mycobacteria. For Hb experiments, lyophilised human Hb (Sigma Aldrich, UK) was reconstituted in RPMI-MGIT and vortexed until a homogenous solution was obtained, which was diluted to the concentration specified. Filtered ferric ammonium citrate (FAC) or deferoxamine (DFO) were diluted in RPMI-MGIT to the concentration specified and warmed for 1 hour at 37 °C. Diluted Hb/FAC/DFO was added to MGIT cultures on day 0 to a total volume of 600 μl per tube. DFO was added to whole blood/PBMC cultures 1 hour prior to addition of mycobacteria to allow time for iron chelation. In all cases, the time to positivity (TTP) was converted to log_10_CFU using stock standard curves of TTP against inoculum volume and CFU. Results are presented as Δ log_10_ CFU (log_10_ CFU of sample − log_10_ CFU of growth control). A titration curve was performed adding serial dilutions of Hb (20 mg/ml to 0.1 mg/ml) directly to Bactec MGIT tubes in the absence of mycobacteria as a negative control; no tubes flagged positive.

### Gene expression microarrays

Microarrays for the infant correlates of risk study were performed as previously described^72^. Briefly, PBMC were thawed and RNA was extracted using an RNeasy kit (Qiagen) according to the manufacturer’s instructions. mRNA was amplified using the Illumina Totalprep kit (Ambion) according to the manufacturer’s instructions, and the quality of the RNA was checked using an Agilent bioanalyser following extraction and again following amplification using the RNA Pico or Nano kits. For whole blood transcriptomic studies, PAXgene^®^ tubes were thawed over two hours at room temperature and total intracellular RNA was extracted using the Blood RNA Kit (Qiagen) according to the manufacturer’s instructions. The purity and quantity of the isolated total RNA was assessed prior to storage at −20 °C until required. Globin mRNA was subsequently depleted using the GLOBINclear Kit (Ambion), amplified and biotin-labelled using the TotalPrep RNA Amplification Kit (Illumina). RNA quality was subsequently assessed. Biotinylated cRNA was hybridised to Illumina HumanHT-12 (v4.0) expression beadchips according to the manufacturer’s instructions. Beadchips were scanned with an Illumina iScan machine, and data extracted using the GenomeStudio software.

### Microarray analysis

Raw, probe level summary values as exported from Illumina GenomeStudio 2011 of Illumina HumanHT 12 V4 microarrays were imported into R using beadarray^73^. Probes were background corrected followed by quantile normalization using the neqc command^74^. The analysis was restricted to probes with a detection p-value of less than 0.01 in at least 10% of the samples and probes matching to GENCODE version 24 with at most 2 mismatches. A linear model was fitted using limma^75^ to determine differential expression including parameters for gender, age, ethnicity and batch effects infection as well as the MGIT growth rate and array quality weights were incorporated^76^ in order to account for between array quality differences. To account for between patient correlation, the duplicateCorrelation command from the limma package was used. All p-values were corrected for multiple hypothesis testing using the Benjamini-Hochberg procedure^77^. Gene set enrichment analysis was carried out using the tmod package in R.

### Statistical analysis

Statistical analysis was performed using GraphPad Prism software Version 5.04 (GraphPad, La Jolla, CA, USA) and SPSS Version 22. The specific test used is indicated in each results section.

## Additional Information

**How to cite this article:** Tanner, R. *et al*. The influence of haemoglobin and iron on *in vitro* mycobacterial growth inhibition assays. *Sci. Rep.* **7**, 43478; doi: 10.1038/srep43478 (2017).

**Publisher's note:** Springer Nature remains neutral with regard to jurisdictional claims in published maps and institutional affiliations.

## Figures and Tables

**Table 1:** Area under the curve is influenced by the addition of Hb, Fe or DFO.

Condition	Mean AUC (Δlog_10_ CFU × weeks)	Corrected p-value (vs. control)
Control	3.28	n/a
Hb added	4.44	0.31
Fe added	5.39	0.20
DFO added	1.61	**0.002

The mean area under the curve (AUC) of BCG Pasteur growth in the MGIT assay during the course of a longitudinal study of 7 macaques using normal blood (control) or blood with haemoglobin (Hb), ferric ammonium citrate (FAC) or the iron chelator deferoxamine (DFO) added. A paired t-test was performed on the AUC between each condition and the control, where **represents a p value of <0.005.

**Table 2:** Correlations between Hb gene expression and MGIT mycobacterial growth.

Description	UK/Asian adults (n = 21) Whole Blood MGIT			South African infants (n = 128) PBMC MGIT		
	logFC	p-value	adj. p-val	logFC	p-value	adj. p-val
alpha haemoglobin stabilizing protein	2.04	*2.67E-02	1.05E-01	0.16	4.91E-01	6.88E-01
cytochrome b5 reductase 3	−0.34	2.52E-01	4.60E-01	0.01	8.54E-01	9.30E-01
haemoglobin, alpha 1 and 2	0.15	6.50E-01	8.03E-01	1.53	*2.22E-02	1.01E-01
haemoglobin, beta	0.08	7.36E-01	8.58E-01	1.47	*2.19E-02	*9.98E-02
haemoglobin, delta	2.54	1.13E-01	2.75E-01	0.73	*3.54E-02	1.35E-01
haemoglobin, epsilon 1	−0.74	6.35E-01	7.93E-01	ND	ND	ND
haemoglobin, gamma A	1.81	1.06E-01	2.65E-01	1.87	*7.10E-03	*4.94E-02
haemoglobin, gamma G	1.78	1.15E-01	2.78E-01	1.78	*7.66E-03	*5.17E-02
haemoglobin, mu	1.87	*3.93E-02	1.37E-01	0.36	1.95E-01	3.96E-01
haemoglobin, theta 1	1.76	*5.62E-02	1.75E-01	0.17	3.35E-01	5.48E-01
haemoglobin, zeta	2.08	4.29E-01	6.35E-01	ND	ND	ND

mycobacterial growth was treated as a continuous variable from two different studies of whole blood from 21 healthy adults from the UK and Asia in an *M.tb* H37Rv-stimulated MGIT PBMC and from 130 South African infants in a BCG-stimulated MGIT assay. Gene expression levels were taken from unstimulated samples for individual genes from the GO category ‘haemoglobin complex’. logFC refers to the amount by which gene expression increases per unit increase in mycobacterial growth. *Indicates significant correlations (p < 0.05). ND = not detected.
