# Author Correction: The influence of haemoglobin and iron on *in vitro* mycobacterial growth inhibition assays

**DOI:** 10.1038/s41598-019-41387-8

**Published:** 2019-04-03

**Authors:** Rachel Tanner, Matthew K. O’Shea, Andrew D. White, Julius Müller, Rachel Harrington-Kandt, Magali Matsumiya, Mike J. Dennis, Eneida A. Parizotto, Stephanie Harris, Elena Stylianou, Vivek Naranbhai, Paulo Bettencourt, Hal Drakesmith, Sally Sharpe, Helen A. Fletcher, Helen McShane

**Affiliations:** 1 The Jenner Institute, University of Oxford, Oxford, UK; 2 Public Health England, Porton Down, Salisbury, UK; 3 Weatherall Institute of Molecular Medicine, University of Oxford, Oxford, UK; 4 London School of Hygiene and Tropical Medicine, London, UK

Correction to: *Scientific Reports* 10.1038/srep43478, published online 03 March 2017

This Article contains an error in Figure 3b, where the y-axis label ‘Hb (mg/ml)’ is incorrectly given as ‘Hb (mg/dl)’. The correct Figure 3 appears below as Figure 1.

**Figure 1:**
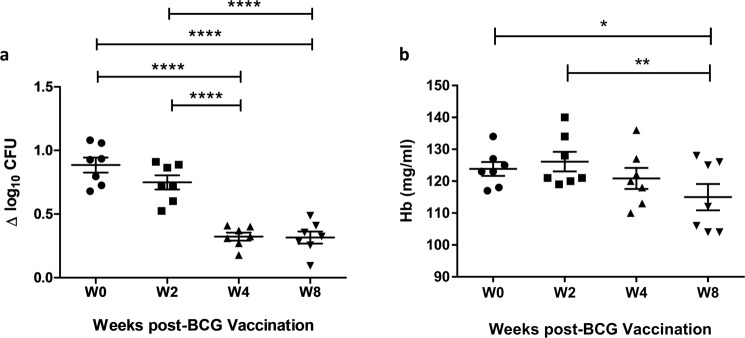
Reduction in Mycobacterial growth and Hb concentration following BCG vaccination and successive bleeds in Rhesus macaques. (**a**) The MGIT assay was performed using BCG Pasteur and (**b**) Hb measured pre- and post-BCG vaccination using whole blood from 7 Rhesus macaques. Points represent the mean of duplicates from individual animals and bars represent the mean values with SEM. Shapes represent different time-points. Having passed a normality test, a repeated measures ANOVA was performed followed by a Bonferroni post-test where ^*^represents a p-value of <0.05, ^**^represents a p value of <0.005, and ^****^represents a p-value of <0.0001. Δ log_10_ CFU = (log_10_ CFU of sample − log_10_ CFU of control).

